# Enhancement by Wy-14,643, a hepatic peroxisome proliferator, of diethylnitrosamine-initiated hepatic tumorigenesis in the rat.

**DOI:** 10.1038/bjc.1978.241

**Published:** 1978-10

**Authors:** J. K. Reddy, M. S. Rao

## Abstract

**Images:**


*Br. J. Cancer* (1978) **38**, 537

### ENHANCEMENT BY WY-14,643, A HEPATIC PEROXISOME PROLIFERATOR, OF DIETHYLNITROSAMINE-INITIATED HEPATIC TUMORIGENESIS IN THE RAT

J. K. REDDY AND M. S. RAO

*From the Department of Pathology, Northwestern University Medical School, 303 East Chicago Avenue, Chicago, Illinois* 60611, *U.S.A.*

Received 11 July 1978 Accepted 25 July 1978

**Summary.—Diethylnitrosamine (DEN), at a concentration of 100 parts/10⁶ in drinking water for 14 days, caused the development, by 48 weeks, of very few liver tumours in 5 of 18 (27%) male F-344 rats fed control diet. When the DEN treatment was followed one week later by continuous feeding of the hypolipidemic hepatic peroxisome proliferator, Wy-14,643, at 0·1% dietary level, all of 28 rats (100%) developed, between 38 and 48 weeks, a significantly higher number of liver tumours. Furthermore, laparotomy at 22 weeks revealed that several rats fed Wy-14,643 after DEN initiation had developed visible liver nodules, suggesting that Wy-14,643 also accelerates the appearance of these tumours. Administration of another peroxisome proliferator, clofibrate, at 0·5% level in the diet after DEN initiation, also caused a substantial enhancement of liver tumorigenesis. The enhancement of liver-tumour development by clofibrate, however, was less than that by Wy-14,643. The marked enhancing effect of Wy-14,643 may be due to its profound hepatomegalic and peroxisome proliferative properties.**

CHEMICAL carcinogens, even when administered at a subcarcinogenic level, produce an irreversible neoplastic change in some cells of the target organ which is referred to as "initiation". Subsequent exposure to certain non-carcinogenic or weakly carcinogenic agents leads to accelerated development of tumours, a process referred to as "promotion" (Boutwell, 1974; Frei, 1976). This concept of initiation and promotion, was first defined for skin carcinogenesis and forms the basis of the "two stage hypothesis" (Berenblum & Shubik, 1947; Salaman & Roe, 1964). Interest in recent years has been increasingly focused on the important problem of identifying factors that modify the "initiation and promotion" phases of carcinogenesis in various organs.

It is now clearly established that the number of tumours in skin or in liver is increased when the cells are stimulated to proliferate before the administration of the carcinogen, suggesting that rapidly proliferating cells are more susceptible to the initiating effect of a carcinogen (Glinos *et al.*, 1951; Pound & Withers, 1963; Frei & Harsono, 1967; Hollander & Bentvelzen, 1968; Svoboda & Reddy, 1970; Craddock, 1971; Reddy *et al.*, 1976*a*; Pound & McGuire, 1978*a*). Furthermore, cell proliferation also appears to play an important part in the promotion of carcinogenesis, especially in skin (Boutwell, 1974; Frei, 1976; Yuspa *et al.*, 1976; Slaga *et al.*, 1976).

Peraino *et al.* (1971; 1977) reported that feeding a diet containing phenobarbital to rats previously fed the hepatocarcinogen, 2-aceylaminofluorene for a brief period, markedly increased the subsequent incidence of hepatic tumour, suggesting that the stages of initiation and promotion are also operable in liver tumorigenesis. Subsequent work by Weisburger *et al.* (1975) confirmed the tumour-promoting effect of phenobarbital in rat liver, though the mechanism of phenobarbital promotion remains unknown. Phenobarbital is a widely used drug which causes hepatomegaly, due to increased cell division and proliferation of smooth endoplasmic reticulum in liver cells, as well as inducing drug-metabolizing microsomal enzymes (Conney, 1967; Schulte-Hermann, 1974). In recent years, an increasing array of natural and synthetic chemicals such as pesticides, drugs, food additives and other xenobiotics has been shown to induce hepatomegaly and enzyme induction similar to that due to phenobarbital treatment (Barka and Popper, 1967; Shulte-Herman, 1974). Since these agents are potential environmental contaminants, it would be important to assess their role as promoters of liver carcinogenesis, even if they are found to be non-carcinogenic.

We have shown that several hypolipidemic agents, when administered to rats and mice, produce a profound hepatomegalic effect associated with proliferation of peroxisomes in liver cells (Reddy *et al.*, 1974; Reddy & Krishnakantha, 1975). Since these agents are administered clinically for prolonged periods, for the control of hyperlipidemia, delineation of various long-term effects of persistent peroxisome proliferation and hepatomegaly appears necessary. Long-term treatment with one of these agents, nafenopin, a closely related analogue of the hypolipidemic drug clofibrate, caused the development of hepatocellular carcinomas in mice and rats (Reddy *et al.*, 1976*b*; Reddy & Rao, 1977).

We now report that Wy-14,643 ([4-chloro-6-(2,3-xylidino)-2-pyrimidinylthio] acetic acid), a potent hepatic peroxisome proliferator (Reddy & Krishnakantha, 1975) when administered to rats after a 14-day initiation with the carcinogen diethylnitrosamine, enhances the development of liver tumours in rats. A comparison has also been made with the promoting effects of clofibrate on liver tumorigenesis in rats similarly exposed to diethylnitrosamine.

### MATERIALS AND METHODS

*Animals and chemicals.*—Inbred male F-344 rats weighing 135–150 g, obtained from Charles River, Wilmington, Mass. U.S.A., were housed in individual cages. Wy-14,643 ([4-chloro-6-(2,3-xylidino)-2-pyrimidinylthio)] acetic acid) was supplied generously by Dr R. M. Tomarelli, Wyeth Laboratories, Inc., Radnor, Pa. U.S.A. Clofibrate (ethyl-α-p-chlorophenoxyisobutyrate) was provided generously by Ayerst Laboratories Inc., New York, N.Y., U.S.A. Diethylnitrosamine (N-nitrosodiethylamine) was obtained from Eastman Kodak Co., Rochester, N.Y. U.S.A.

*Diethylnitrosamine administration.* — Diethylnitrosamine (DEN) was incorporated into drinking water freshly each day at a concentration of 100 parts/$10^6$ and given for 14 days. After this initiation period, the DEN administration was discontinued and the animals were fed control diet and water *ad libitum*, for 7 days. After this recovery period, the animals were divided into 3 groups to study the effect of Wy-14,643 and clofibrate on the post-initiation (promotion) phase of liver tumorigenesis as described below.

*Administration of Wy-*14,643 *and clofibrate after DEN initiation.*—The three groups of DEN-initiated rats were treated as follows: Group 1, of 30 rats, was fed Wy-14,643 at a dietary concentration of 0·1%; Group 2, of 30 rats, was fed clofibrate at a dietary level of 0·5%, and Group 3, of 20 rats, was fed the basic diet of Purina Laboratory Chow (Ralston Purina Company, St Louis, Mo., U.S.A.) until the termination of the experiment at after 48 weeks of these dietary regimes. Animals were observed carefully and weighed once a week. Laparotomies were performed at 22 weeks to assess the development of liver tumours. Two animals from each group were killed at this time for histological studies. The experiment was terminated at 48 weeks, because of progressive loss of weight and deaths due to liver tumours occurring in rats fed Wy-14,643 between 38 and 48 weeks. Animals were killed by ether inhalation. Autopsies were done and liver, lungs and kidneys were fixed in 10% neutral buffered formalin. Histological sections of selected lesions were stained routinely with hematoxylin and eosin.

### RESULTS

All rats survived the 14-day treatment with DEN in drinking water. Laparotomies performed at 22 weeks revealed the presence of one or more grossly visible nodules 2–6 mm in diameter in the livers of ~60% of rats fed Wy-14,643 after DEN initiation. In contrast, only an occasional rat fed clofibrate, and none of the rats on control diet, after DEN initiation, had grossly visible lesions at 22 weeks. Histological examination of the livers of rats killed at this time (2 animals from each group) showed hyperplastic nodules and many foci of liver-cell proliferation in only the Wy-14,643 group (Fig. 1).

Between 38 and 48 weeks, 5/28 rats fed Wy-14,643 after DEN initiation, died with liver tumours. As these animals lost considerable weight, all surviving rats were killed at the 48th week and examined for hepatic tumours. The Table compares the liver tumour incidence in the 3 groups. The data indicate that 5/18 rats (27%) fed control diet after DEN initiation developed liver tumours. A total of 7 tumours were found in these 5 livers, only 3 of which were 10–15 mm in diameter. In contrast, all 28 rats (100%) fed 0·1% Wy-14,643 after DEN initiation, devel-

FIG. 1.—Liver from a rat fed 0·1% Wy-14,643 in the diet for 22 weeks after DEN initiation. Hyperplastic liver nodule. H. and E. ×100.

TABLE.—*Liver-tumour incidence in F-*344 *male rats fed Wy-*14,643 *or* 0·5% *clofibrate* (*CPIB*) *for* 48 *weeks from* 7 *days after the administration of diethylnitrosame* (*DEN*). *DEN was added to the drinking water at a concentration of* 100 *parts*/10⁶ *during the* 14-*day initiation period.*

| Treatment after DEN initiation | No. of rats‡ | No. with liver tumours | % with liver tumours | No. of tumours >0.5 cm/ liver§ | No. of tumours >1 cm/ liver§ |
|---|---|---|---|---|---|
| Control diet | 18 | 5 | 27 | 0·38 | 0·16 |
| 0.1% Wy-14,643 | 28 | 28† | 100* | 9·50* | 5·00* |
| 0.5% CPIB | 28 | 25 | 89* | 1·50 | 0·53 |

* Significantly different from rats fed control diet as determined by $\chi^2$ test with 1 degree of freedom ($P<0.001$).
† Two of these rats also had renal-cell adenomas measuring about 7 mm in diameter.
‡ Excluding rats killed at 22 weeks (2 from each group).
§ In relation to the total number of rats.

FIG. 2.—Comparison of 4 randomly selected livers from control and Wy-14,643-fed groups after DEN initiation and killed at 48 weeks. Note the presence of numerous tumours in Wy-14,643-fed group.

oped multiple liver tumours, 2–35 mm in diameter, involving all lobes (Fig. 2). For convenience, the data in the Table deal with tumours over 5 mm in diameter. The number of nodules under 5 mm in diameter, in the livers of animals fed Wy-14,643, were too numerous to arrive at a meaningful estimate. Histologically, all the tumours were hepatocellular in origin and those larger than 10 mm in diameter were hepatocellular carcinomas (Fig. 3). Two rats fed Wy-14,643 had clear-cell adenomas of the kidney measuring about 7 mm in diameter. In rats fed clofibrate after DEN initiation, 25/28 animals (89%) showed liver tumours. The overall tumour incidence in the clofibrate group was significantly greater than that in the control group, but was significantly less than that in the Wy-14,643 group. In animals fed control diet after DEN initiation, only 3 hepatocellular carcinomas were found, but several livers contained an occasional microscopic focus of altered liver cells (Fig. 4), suggesting tumour foci, that were either dormant or proliferating very slowly.

### DISCUSSION

Of several factors that appear to promote the chemical carcinogen-initiated development of liver tumours (Glinos *et al.*, 1951; Hollander & Bentvelzen, 1968; Svoboda & Reddy, 1970; Pound & McGuire, 1978*a* and *b*; Feinstein *et al.*, 1978), phenobarbital (Peraino *et al.*, 1971) and dichlorodiphenyltrichloroethane (DDT) (Peraino *et al.*, 1975) appear unique in that they produce hepatomegaly and stimulate DNA synthesis without causing liver-cell necrosis. Furthermore, both phenobarbital and DDT are potent inducers of smooth endoplasmic reticulum proliferation in liver cells, which is accompanied by an increase in liver microsomal enzymes (Conney, 1967; Schulte-Hermann, 1974). The results of the present study demonstrate that prolonged dietary administration of 2 hepatic peroxisome proliferators, Wy-14,643 and clofibrate, significantly enhance the development of liver tumours in rats previously exposed to DEN for a brief period. In addition to enhanced incidence, the number of hepatocellular tumours that developed in each rat,

FIG. 3.—Hepatocellular carcinoma from a rat fed Wy-14,643 for 48 weeks after DEN initiation. Shows trabecular pattern. H. and E. ×200

FIG. 4.—Liver of a rat fed control diet for 48 weeks after DEN initiation. Focal proliferations of liver cells such as these indicate slow or arrested growth of initiated cell(s). H. and E. ×100.

especially with Wy-14,643 feeding, was also greatly increased. Continuous feeding of these peroxisome proliferators, therefore, appears to act as a promotor of liver carcinogenesis.

Wy-14,643 and clofibrate are known to reduce serum lipids, and cause a marked increase in liver weight in both rats and mice (Hess *et al.*, 1965; Svoboda *et al.*, 1967; Reddy & Krishnakantha, 1975). In addition, they induce the proliferation of peroxisomes in liver cells, with an increase in some enzymes associated with this organelle (Reddy & Krishnakantha, 1975; Moody & Reddy, 1978). These changes are considered analogous to the proliferation of smooth endoplasmic reticulum and induction of microsomal enzymes in liver by phenobarbital and other xenobiotics. Because of the long-term administration of drugs such as these for the control of hyperlipidemic states in man, it is essential to investigate various aspects of persistent hepatomegaly and peroxisome proliferation.

The mechanism of enhancement of liver tumorigenesis in animals fed Wy-14,643 or clofibrate, after DEN initiation remains to be elucidated. All potent peroxisome proliferators such as Wy-14,643, BR-931 and nafenopin (Reddy *et al.*, 1978*b*; Moody *et al.*, 1977) stimulate liver-cell proliferation during the initial stages of feeding, as well as causing cellular hypertrophy. The hepatomegalic as well as mitogenic effects of clofibrate appear somewhat less pronounced than with Wy-14,643 (Moody & Reddy, 1978). The question arises whether the increased hepatocellular tumour yield in Wy-14,643-fed rats, over the clofibrate-fed group, reflects the mitogenic potency of this agent. Several studies have stressed that cell proliferation is important in the promotion of skin carcinogenesis (Boutwell, 1974; Frei, 1976) and it is possible that phenobarbital (Peraino *et al.*, 1971) DDT (Peraino *et al.*, 1975) carbon tetrachloride (Pound & McGuire, 1978*b*) and now the peroxisome proliferators, exert their enhancing effect on liver tumorigenesis by increasing the rate of liver-cell proliferation. In addition, drugs such as phenobarbital, DDT, Wy-14,643 and clofibrate increase the membranes in liver cells, and may influence membrane functions. The role, if any, of persistent increase in peroxisomes and/or smooth endoplasmic reticulum in liver-tumour promotion needs to be clarified. Furthermore, emerging evidence indicates that potent peroxisomes proliferators such as nafenopin, a closely related analogue of clofibrate, and Wy-14,643, which is structurally unrelated to clofibrate, are hepatocarcinogenic to rats and mice when administered for prolonged periods (Reddy *et al.*, 1976*b*; Reddy & Rao, 1977; Reddy *et al.*, 1978*a*; Reddy, unpublished). Unpublished data also suggest that the drug clofibrate may be a weak hepatocarcinogen. These observations, coupled with the data presented in this paper on the enhancing effect of Wy-14,643 and clofibrate on liver-tumour development in rats after DEN initiation, suggest the need for long-term studies with other hypolipidemic peroxisome proliferators to clarify the role of peroxisomes in hepatocarcinogenesis and/or in its promotion.

This work was supported by USPHS Grant GM 23750 from the National Institute of General Medical Sciences.

REFERENCES

Barka, T. & Popper, H. (1967) Liver enlargement and drug toxicity. *Medicine (Baltimore)*, **46**, 103.

Berenblum, I. & Shubik, P. (1947) A new quantitative approach to the study of the stages of chemical carcinogenesis in the mouse's skin. *Br. J. Cancer*, **1**, 383.

Boutwell, R. K. (1974) The function and mechanism of promoters of carcinogenesis. *CRC Crit. Rev. Toxicol.*, **2**, 419.

Conney, A. H. (1967) Pharmacological implications of microsomal enzyme induction. *Pharmacol. Rev.*, **19**, 317.

Craddock, V. M. (1971) Liver carcinomas induced in rats by single administration of dimethylnitrosamine after partial hepatectomy. *J. Natl. Cancer Inst.*, **47**, 899.

Feinstein, R. N., Fry, R. J. M. & Staffeldt, E. F. (1978) Carcinogenic and antitumor effects of aminotriazole on acatalasemic and normal catalase mice. *J. Natl. Cancer Inst.*, **60**, 1113.

Frei, J. V. & Harsono, T. (1967) Increased susceptibility to low doses of a carcinogen of epidermal cells in stimulated DNA synthesis. *Cancer Res.*, **27**, 1482.

FREI, J. V. (1976) Some mechanisms operative in carcinogenesis, a review. *Chem. Biol. Interact.*, **13**, 1.

GLINOS, A. D., BUCHER, N. L. R. & AUB, J. C. (1951) The effect of liver regeneration on tumour formation in rats fed 4-dimethylaminoazobenzene. *J. Exp. Med.*, **93**, 313.

HESS, R., STAÜBLI, W. & REISS, W. (1965) Nature of the hepatomegalic effect produced by ethylchlorophenoxyisobutyrate in the rat. *Nature*, **208**, 856.

HOLLANDER, C. F. & BENTVELZEN, P. (1968) Enhancement of urethan induction of hepatomas in mice by prior partial hepatectomy. *J. Natl. Cancer Inst.*, **41**, 1303.

MOODY, D. E., RAO, M. S. & REDDY, J. K. (1977) Mitogenic effect in mouse liver induced by a hypolipidemic drug, nafenopin. *Virchows Arch. B. (Cell Pathol.)*, **23**, 291.

MOODY, D. E. & REDDY, J. K. (1978) The hepatic effects of hypolipidemic drugs (clofibrate, nafenopin, tibric acid and Wy-14,643) on hepatic peroxisomes and peroxisome-associated enzymes. *Am. J. Pathol.*, **90**, 435.

PERAINO, C., FRY, R. J. M. & STAFFELDT, E. (1971) Reduction and enhancement by phenobarbital of hepatocarcinogenesis induced in the rat by 2-acetylaminofluorene. *Cancer Res.*, **31**, 1506.

PERAINO, C., FRY, R. J. M. & STAFFELDT, E. (1977) Effects of varying the onset and duration of exposure to phenobarbital on its enhancement of 2-acetylaminofluorene-induced hepatic tumorigenesis. *Cancer Res.*, **37**, 3623.

PERAINO, C., FRY, R. J. M., STAFFELDT, E. & CHRISTOPHER, J. P. (1975) Comparative enhancing effects of phenobarbital, amobarbital, diphenylhydantoin, and dichlorodiphenyltrichloroethane on 2-acetylaminofluorene-induced hepatic tumourigenesis in the rat. *Cancer Res.*, **35**, 2884.

POUND, A. W. & MCGUIRE, L. J. (1978*a*) Repeated partial hepatectomy as a promoting stimulus for carcinogenic response of liver to nitrosamines in rats. *Br. J. Cancer*, **37**, 585.

POUND, A. W. & MCGUIRE, L. J. (1978*b*) Influence of repeated liver regeneration on hepatic carcinogenesis by diethylnitrosamine in mice. *Br. J. Cancer*, **37**, 595.

POUND, A. W. & WITHERS, H. R. (1963) The influence of some irritant chemicals and scarification on tumour irritation by urethane in mice. *Br. J. Cancer*, **17**, 460.

REDDY, J. K., AZARNOFF, D. L., RAO, M. S., & QURESHI, S. A. (1978*a*) Hepatocarcinogenicity of Wy-14,643, a hypolipidemic peroxisome proliferator. (Abst.). *Fed. Proc.*, **37**, 232.

REDDY, J. K., AZARNOFF, D. L. & SIRTORI, C. R. (1978*b*) Hepatic peroxisome proliferation: induction by BR-931, a hypolipidemic analog of Wy-14,643. *Arch. Int. Pharmacodyn.* (in press).

REDDY, J. K., AZARNOFF, D. L., SVOBODA, D. J. & PRASAD, J. D. (1974) Nafenopin induced hepatic microbody (peroxisome) proliferation and catalase synthesis in rats and mice. Absence of sex difference in response. *J. Cell Biol.*, **61**, 344.

REDDY, J. K. & KRISHNAKANTHA, T. P. (1975) Hepatic peroxisome proliferation: induction by two novel compounds structurally unrelated to clofibrate. *Science*, **190**, 787.

REDDY, J. K. & RAO, M. S. (1977) Malignant tumors in rats fed nafenopin, a hepatic peroxisome proliferator. *J. Natl. Cancer Inst.*, **59**, 1645.

REDDY, J. K., RAO. M. S. & JAGO, M. V. (1976*a*) Rapid development of hyperplastic nodules and cirrhosis in the liver of rats treated concurrently with thioacetamide and the pyrrolizidine alkaloid lasiocarpine. *Int. J. Cancer*, **17**, 621.

REDDY, J. K., RAO, M. S. & MOODY, D. E. (1976*b*) Hepatocellular carcinomas in acatalasemic mice treated with nafenopin, a hypolipidemic peroxisome proliferator. *Cancer Res.*, **36**, 1211.

SALAMAN, M. H. & ROE, F. J. C. (1964) Cocarcinogenesis. *Br. Med. Bull.*, **20**, 139.

SCHULTE-HERMANN, R. (1974) Induction of liver growth by xenobiotic compounds and other stimuli. *CRC Crit. Rev. Toxicol.*, **3**, 97.

SLAGA, T. J., SCRIBNER, J. D., THOMPSON, S. & VIAJE, A. (1976) Epidermal cell proliferation and promoting ability of phorbol esters. *J. Natl. Cancer Inst.*, **57**, 1145.

SVOBODA, D. & REDDY, J. (1970) Some effects of carcinogens on the structure and activity of liver cells In *Metabolic Aspects of Food Safety*. Ed F. J. C. Roe. Oxford: Blackwell, p. 533.

SVOBODA, D., GRADY, H. & AZARNOFF, D. (1967) Microbodies in experimentally altered cells. *J. Cell Biol.*, **35**, 127.

WEISBURGER, J. H., MADISON, R. M., WARD, J. M., VIGUERA, C. & WEISBURGER, E. K. (1975) Modification of diethylnitrosamine liver carcinogenesis with phenobarbital but not with immunosuppression. *J. Natl. Cancer Inst.*, **54**, 1185.

YUSPA, S. H., HENNINGS, H. & SAFFIOTI, U. (1976) Cutaneous chemical carcinogenesis: past, present and future. *J. Invest. Dermatol.*, **67**, 199.


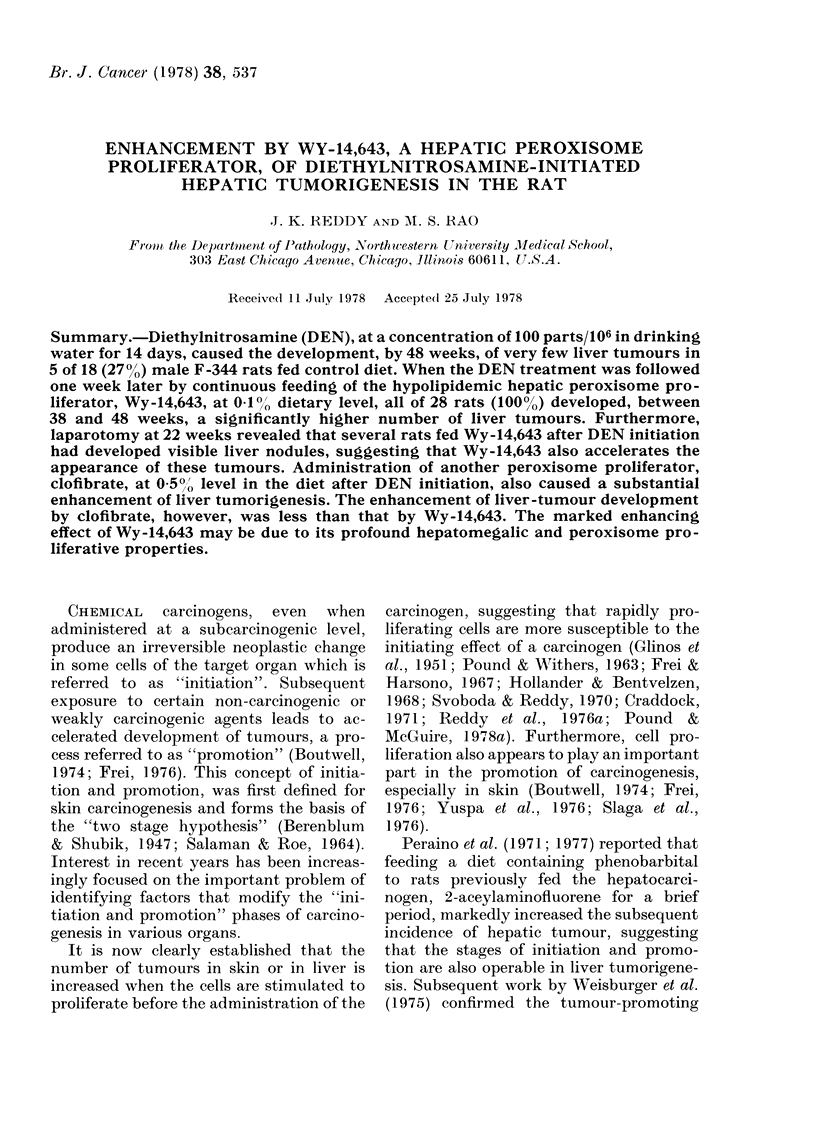

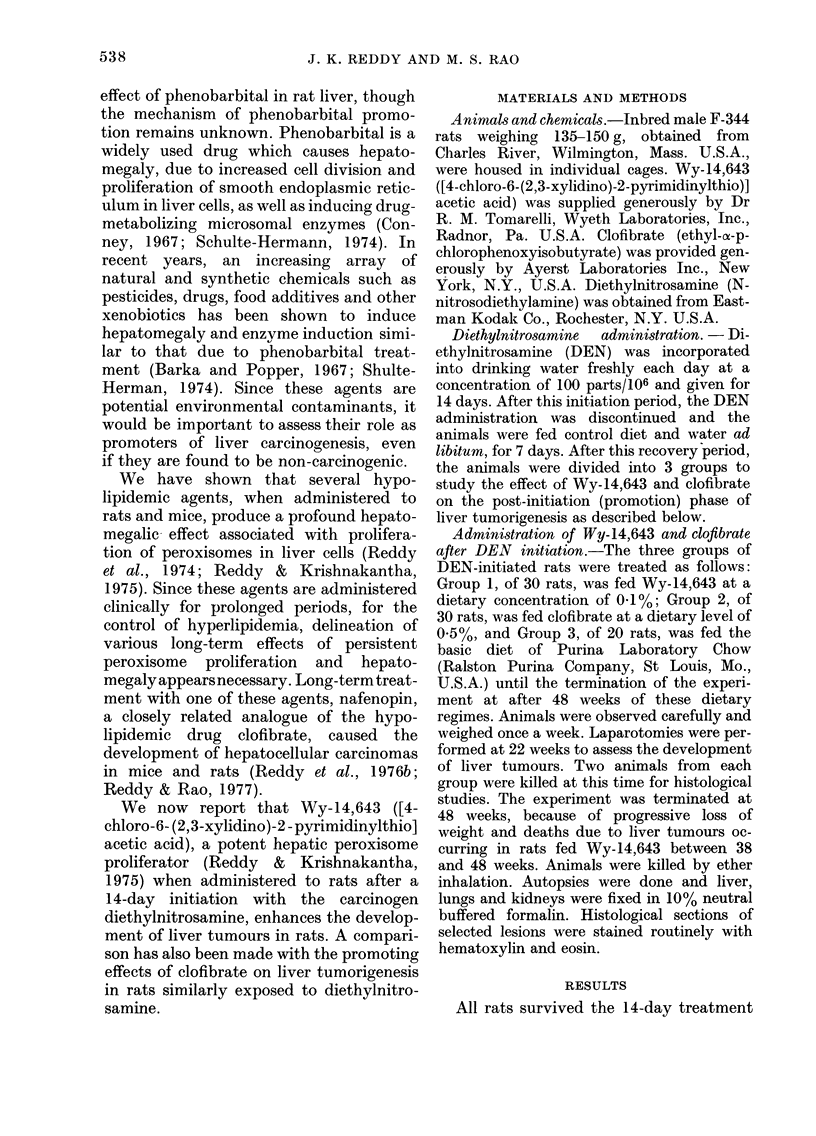

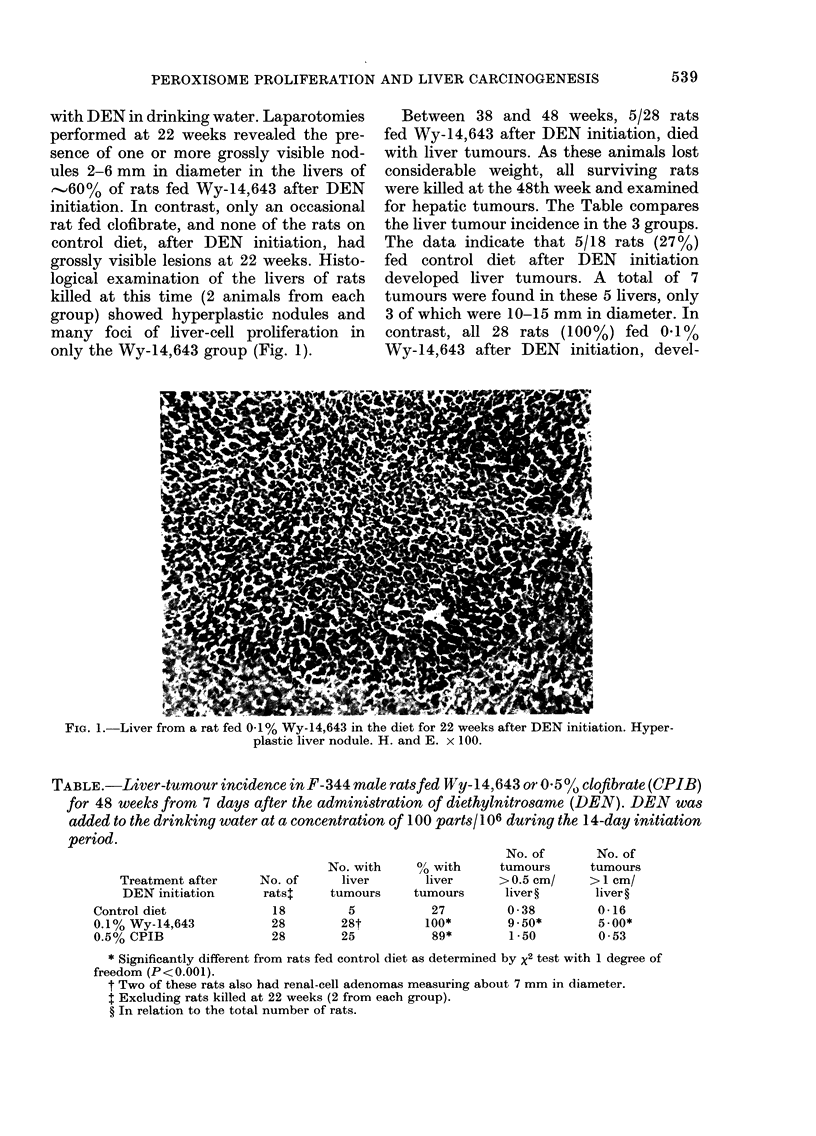

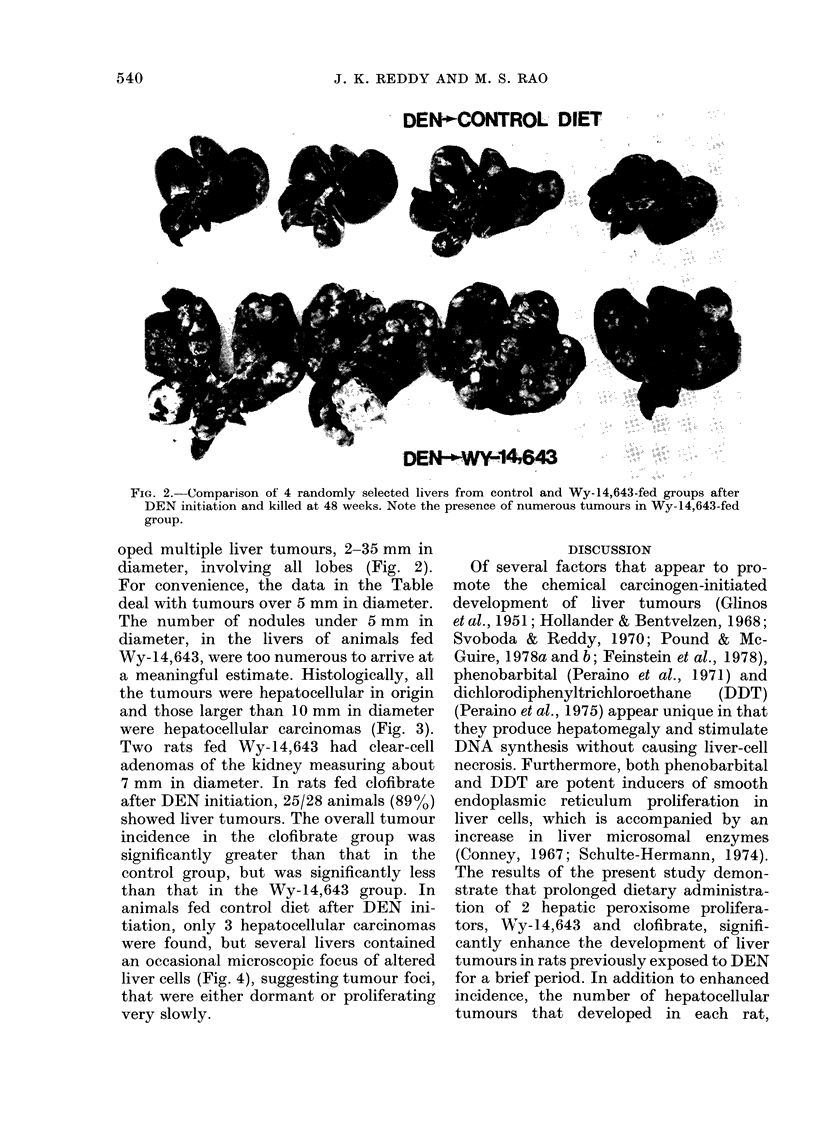

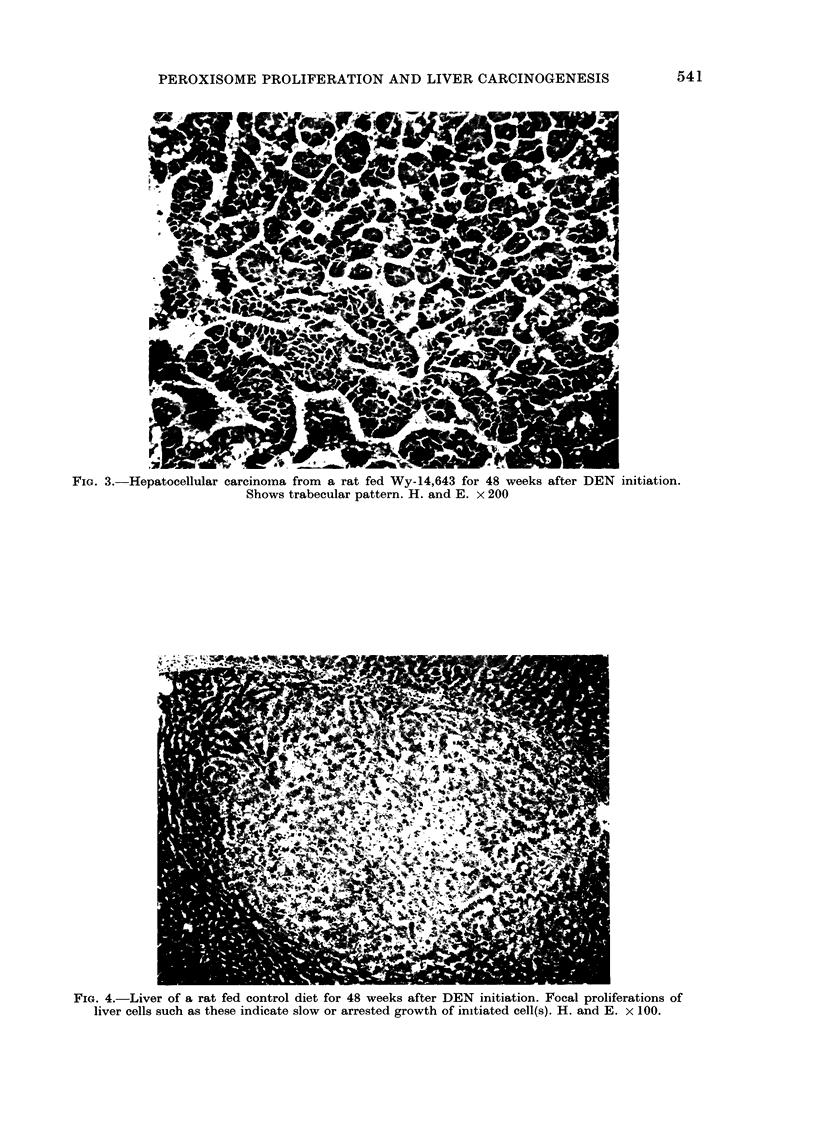

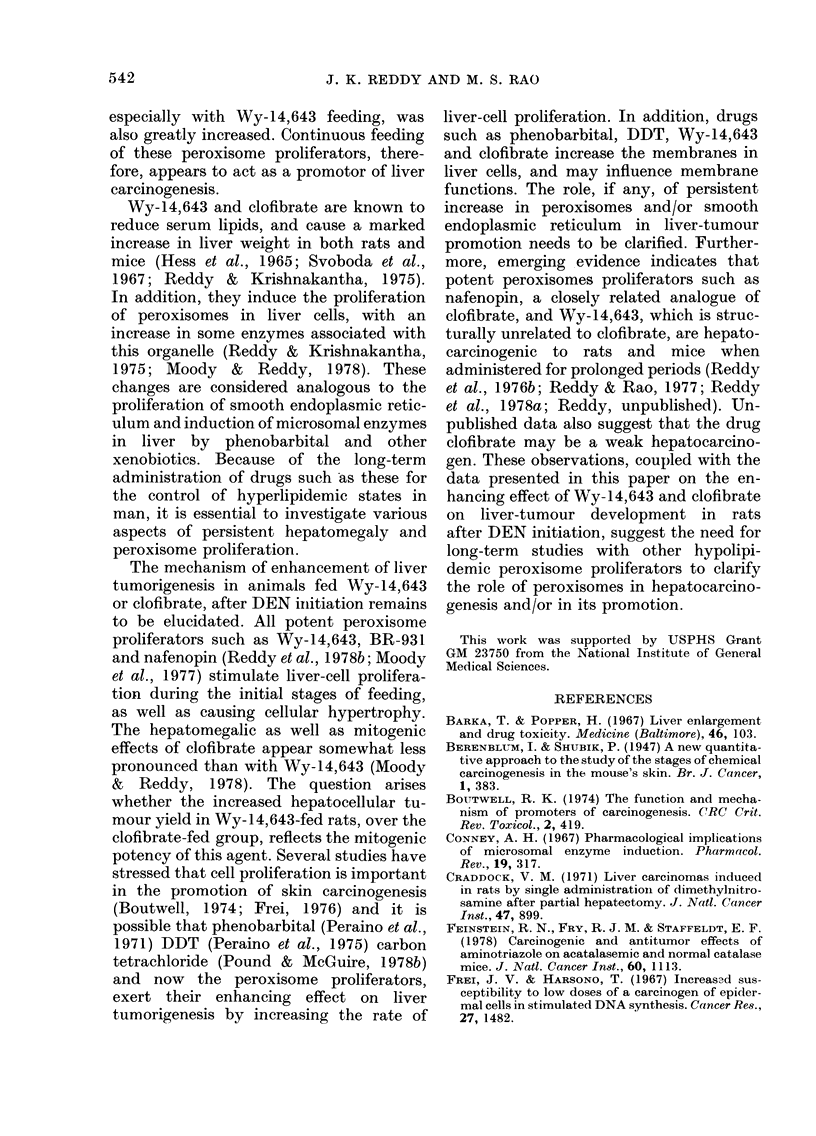

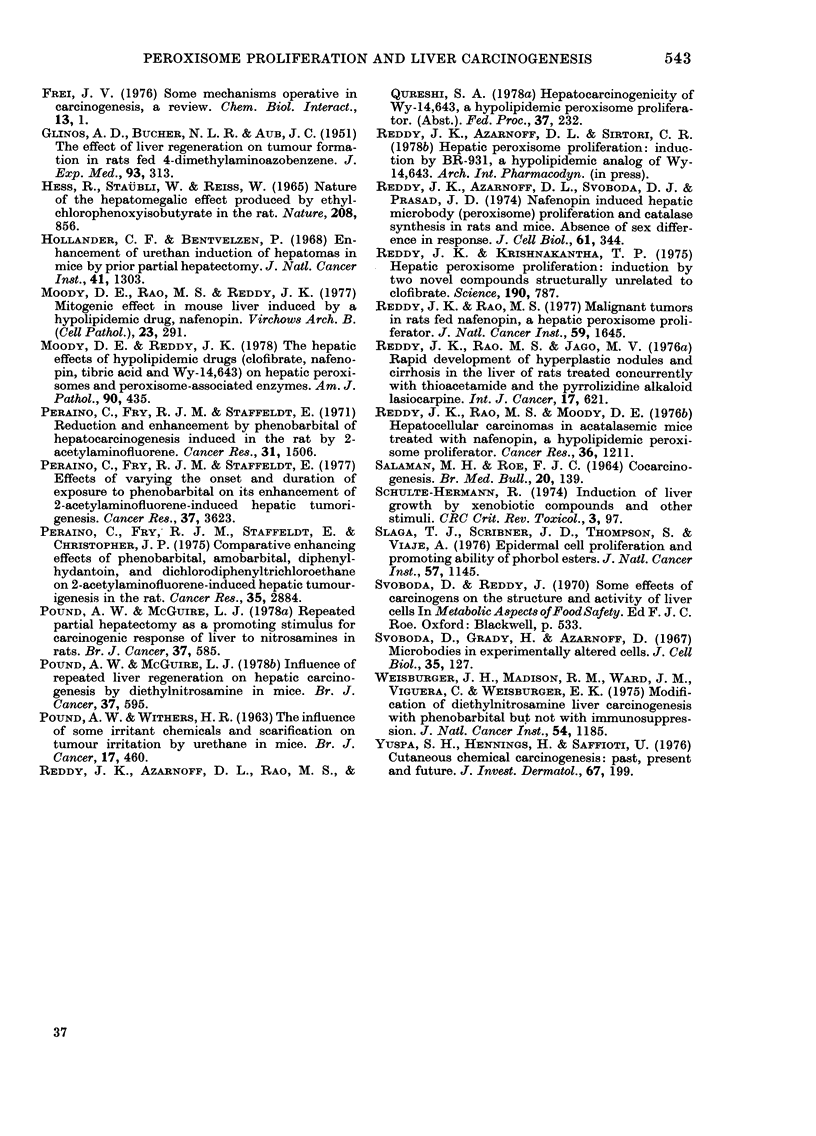


## References

[OCR_00438] Barka T., Popper H. (1967). Liver enlargement and drug toxicity.. Medicine (Baltimore).

[OCR_00448] Boutwell R. K. (1974). The function and mechanism of promoters of carcinogenesis.. CRC Crit Rev Toxicol.

[OCR_00453] Conney A. H. (1967). Pharmacological implications of microsomal enzyme induction.. Pharmacol Rev.

[OCR_00458] Craddock V. M. (1971). Liver carcinomas induced in rats by single administration of dimethylnitrosamine after partial hepatectomy.. J Natl Cancer Inst.

[OCR_00464] Feinstein R. N., Fry R. J., Staffeldt E. F. (1978). Carcinogenic and antitumor effects of aminotriazole on acatalasemic and normal catalase mice.. J Natl Cancer Inst.

[OCR_00470] Frei J. V., Harsono T. (1967). Increased susceptibility to low doses of a carcinogen of epidermal cells in stimulated DNA synthesis.. Cancer Res.

[OCR_00478] Frei J. V. (1976). Some mechanisms operative in carcinogenesis a review.. Chem Biol Interact.

[OCR_00483] GLINOS A. D., BUCHER N. L. R., AUB J. C. (1951). The effect of liver regeneration on tumor formation in rats fed 4-dimethylaminoazobenzene.. J Exp Med.

[OCR_00489] Hess R., Stäubli W., Riess W. (1965). Nature of the hepatomegalic effect produced by ethyl-chlorophenoxy-isobutyrate in the rat.. Nature.

[OCR_00495] Hollander C. F., Bentvelzen P. (1968). Enhancement of urethan induction of hepatomas in mice by prior partial hepatectomy.. J Natl Cancer Inst.

[OCR_00501] Moody D. E., Rao M. S., Reddy J. K. (1977). Mitogenic effect in mouse liver induced by a hypolipidemic drug, nafenopin.. Virchows Arch B Cell Pathol.

[OCR_00507] Moody D. E., Reddy J. K. (1978). The hepatic effects of hypolipidemic drugs (clofibrate, nafenopin, tibric acid, and Wy-14,643) on hepatic peroxisomes and peroxisome-associated enzymes.. Am J Pathol.

[OCR_00547] POUND A. W., WITHERS H. R. (1963). THE INFLUENCE OF SOME IRRITANT CHEMICALS AND SCARIFICATION ON TUMOUR INITIATION BY URETHANE IN MICE.. Br J Cancer.

[OCR_00527] Peraino C., Fry R. J., Staffeldt E., Christopher J. P. (1975). Comparative enhancing effects of phenobarbital, amobarbital, diphenylhydantoin, and dichlorodiphenyltrichloroethane on 2-acetylaminofluorene-induced hepatic tumorigenesis in the rat.. Cancer Res.

[OCR_00520] Peraino C., Fry R. J., Staffeldt E. (1977). Effects of varying the onset and duration of exposure to phenobarbital on its enhancement of 2-acetylaminofluorene-induced hepatic tumorigenesis.. Cancer Res.

[OCR_00514] Peraino C., Fry R. J., Staffeldt E. (1971). Reduction and enhancement by phenobarbital of hepatocarcinogenesis induced in the rat by 2-acetylaminofluorene.. Cancer Res.

[OCR_00541] Poound A. W., McGuire L. J. (1978). Influence of repeated liver regeneration on hepatic carcinogenesis by diethylnitrosamine in mice.. Br J Cancer.

[OCR_00535] Pound A. W., McGuire L. J. (1978). Repeated partial hepatectomy as a promoting stimulus for carcinogenic response of liver to nitrosamines in rats.. Br J Cancer.

[OCR_00565] Reddy J. K., Azarnoff D. L., Svoboda D. J., Prasad J. D. (1974). Nafenopin-induced hepatic microbody (peroxisome) proliferation and catalase synthesis in rats and mice. Absence of sex difference in response.. J Cell Biol.

[OCR_00572] Reddy J. K., Krishnakantha T. P. (1975). Hepatic peroxisome proliferation: induction by two novel compounds structurally unrelated to clofibrate.. Science.

[OCR_00583] Reddy J. K., Rao M. S., Jago M. V. (1976). Rapid development of hyperplastic nodules and cirrhosis in the liver of rats treated concurrently with thioacetamide and the pyrrolizidine alkaloid lasiocarpine.. Int J Cancer.

[OCR_00578] Reddy J. K., Rao M. S. (1977). Malignant tumors in rats fed nafenopin, a hepatic peroxisome proliferator.. J Natl Cancer Inst.

[OCR_00590] Reddy J. K., Rao S., Moody D. E. (1976). Hepatocellular carcinomas in acatalasemic mice treated with nafenopin, a hypolipidemic peroxisome proliferator.. Cancer Res.

[OCR_00596] SALAMAN M. H., ROE F. J. (1964). COCARCINOGENESIS.. Br Med Bull.

[OCR_00600] Schulte-Hermann R. (1974). Induction of liver growth by xenobiotic compounds and other stimuli.. CRC Crit Rev Toxicol.

[OCR_00605] Slaga T. J., Scribner J. D., Viaje A. (1976). Epidermal cell proliferation and promoting ability of phorbol esters.. J Natl Cancer Inst.

[OCR_00617] Svoboda D., Grady H., Azarnoff D. (1967). Microbodies in experimentally altered cells.. J Cell Biol.

[OCR_00622] Weisburger J. H., Madison R. M., Ward J. M., Viguera C., Weisburger E. K. (1975). Modification of diethylnitrosamine liver carcinogenesis with phenobarbital but not with immunosuppression.. J Natl Cancer Inst.

[OCR_00629] Yuspa S. H., Hennings H., Saffiotti U. (1976). Cutaneous chemical carcinogenesis: past, present, and future.. J Invest Dermatol.

